# TP53 Mutation and Tumor‐Infiltrating Lymphocytes as Predictors of PD‐1/PD‐L1 Inhibitor Efficacy in PD‐L1‐High Advanced NSCLC

**DOI:** 10.1002/cnr2.70648

**Published:** 2026-08-13

**Authors:** Hui Yan, Qian Xu, Yushuang Zheng, Meng Shen, Dapeng Li

**Affiliations:** ^1^ Department of Oncology The First Affiliated Hospital of Soochow University Suzhou China; ^2^ Department of Pathology The First Affiliated Hospital of Soochow University Suzhou China

**Keywords:** immune checkpoint inhibitors, nonsmall cell lung cancer (NSCLC), PD‐L1‐high expression, TP53 mutation, tumor‐infiltrating lymphocytes (TILs)

## Abstract

**Background:**

Programmed death‐ligand 1 (PD‐L1) expression is widely used to guide immune checkpoint inhibitor (ICI) therapy in advanced nonsmall cell lung cancer (NSCLC); however, substantial heterogeneity in clinical outcomes persists among patients with high PD‐L1 expression. Reliable biomarkers for further stratifying this population are still lacking. This study investigated whether genomic alterations and features of the tumor immune microenvironment could explain this variability.

**Methods:**

We conducted a retrospective single‐center study including 91 patients with advanced NSCLC and PD‐L1 tumor proportion score (TPS) ≥ 50% who received ICI‐based therapy. Clinical outcomes were assessed according to PD‐L1 expression levels, genomic profiles, and tumor immune microenvironment characteristics. Tumor‐infiltrating lymphocytes (TILs) and tertiary lymphoid structures (TLSs) were evaluated in available tumor specimens from 75 patients. Multiplex immunohistochemistry (mIHC) was performed in a subset of 30 patients to characterize the immune cellular composition underlying TIL‐based stratification. Kaplan–Meier survival analysis and multivariable Cox regression were used to identify factors associated with progression‐free survival (PFS) and overall survival (OS).

**Results:**

At a median follow‐up of 19.1 months, the objective response rate was 31.9%, the median PFS was 10.7 months, and the median OS was 43.5 months. No significant survival differences were observed between the TPS 50%–89% and TPS ≥ 90% subgroups. TP53 mutation was associated with shorter PFS and remained an independent adverse prognostic factor. In contrast, high TIL infiltration was independently associated with improved PFS and OS, and this OS benefit was most pronounced in the TPS ≥ 90% subgroup. mIHC analysis revealed that high‐TIL tumors exhibited increased stromal CD8^+^ T‐cell infiltration, enriched NK‐cell populations, and a more proinflammatory macrophage profile, providing biological support for TIL‐based stratification. TLS presence was not significantly associated with survival outcomes.

**Conclusions:**

PD‐L1 expression alone does not fully account for the outcome heterogeneity observed in PD‐L1‐high NSCLC treated with ICIs. Integrating genomic alterations—particularly TP53 mutation status—with tumor immune microenvironment features, such as TIL density, may help refine prognostic stratification in this population.

## 1 Introduction

Lung cancer remains the most commonly diagnosed cancer and the leading cause of cancer mortality worldwide, accounting for approximately 2.5 million new cases and 1.8 million deaths in 2022 [1]. Nonsmall cell lung cancer (NSCLC) represents approximately 85% of all lung cancers and continues to impose a substantial global health burden [2]. Although conventional chemotherapy and targeted therapies—particularly tyrosine kinase inhibitors (TKIs) targeting epidermal growth factor receptor (EGFR) and anaplastic lymphoma kinase (ALK)—have significantly improved survival outcomes in selected patients, the majority of patients are diagnosed at advanced stages, and durable disease control remains elusive due to acquired resistance and tumor heterogeneity [3, 4].

Immune checkpoint inhibitors (ICIs), specifically those targeting the programmed death 1 (PD‐1) receptor and its ligand PD‐L1, have fundamentally transformed the treatment landscape of advanced NSCLC [5]. Tumor proportion score (TPS), defined as the percentage of viable tumor cells with membranous PD‐L1 staining, remains the most widely used criterion for ICI selection. Clinical evidence has consistently demonstrated that patients with PD‐L1‐high tumors (TPS ≥ 50%) derive substantial benefit from PD‐1/PD‐L1 blockade, with first‐line pembrolizumab achieving a median overall survival (OS) exceeding 30 months and an ORR of approximately 45% in this population. Furthermore, emerging evidence suggests that those with very high PD‐L1 expression (TPS ≥ 90%) may achieve particularly durable responses, with 5‐year survival rates approaching 30% [6, 7]. This heightened sensitivity is biologically plausible: ultra‐high PD‐L1 expression may reflect a greater dependence on the PD‐1/PD‐L1 axis for immune evasion, resulting in tumors that are more reliant on checkpoint signaling [8]. However, PD‐L1 overexpression can also be driven by tumor‐intrinsic oncogenic pathways independent of effective antitumor immunity, potentially contributing to primary resistance even within these highly expressing subgroups [9]. In clinical practice, a subset of PD‐L1‐high patients may not respond favorably to ICIs, highlighting the need for additional biomarkers beyond TPS.

In this study, we investigated the associations between genomic alterations, immune microenvironment features, and clinical outcomes in patients with PD‐L1‐high advanced NSCLC receiving ICI‐based therapy. We focused on recurrent genomic alterations (TP53, KRAS, MET, and EGFR) and routinely assessable histopathological immune biomarkers, including tumor‐infiltrating lymphocytes (TILs) and tertiary lymphoid structures (TLSs). Furthermore, multiplex immunohistochemistry (mIHC) was performed to characterize immune cell composition associated with TIL‐based stratification. By integrating these features, we aimed to refine prognostic stratification beyond PD‐L1 expression alone and further elucidate the biological heterogeneity underlying variable immunotherapy responses in PD‐L1‐high NSCLC.

## 2 Methods

### 2.1 Study Population and Design

This retrospective single‐center cohort study included patients with advanced NSCLC who received ICI‐based therapy at the First Affiliated Hospital of Soochow University between November 2019 and October 2024. Eligible patients met the following criteria: (i) histologically or cytologically confirmed NSCLC; (ii) PD‐L1 TPS ≥ 50%; (iii) unresectable stage III or stage IV disease (AJCC 8th edition); (iv) treatment with PD‐1/PD‐L1 inhibitor therapy in any line, alone or combined with chemotherapy and/or targeted therapy; and (v) availability of complete clinical, pathological, and follow‐up data. Patients were excluded if they had a history of other active malignancies, ongoing systemic immunosuppressive therapy, or incomplete clinical or follow‐up data.

As this was a real‐world observational cohort, patients received heterogeneous ICI‐based regimens, including monotherapy, chemo‐immunotherapy, and antiangiogenic combinations, according to treating physicians' decisions and contemporary clinical guidelines. Chemotherapy backbones included platinum‐based doublets (e.g., pemetrexed, paclitaxel, gemcitabine, docetaxel, and albumin‐bound paclitaxel), administered at standard doses per institutional practice and published guidelines. A total of 91 patients were included in the final analysis. The study protocol was approved by the Ethics Committee of the First Affiliated Hospital of Soochow University (Approval No. 2025347) and was conducted in accordance with the Declaration of Helsinki.

### 2.2 Data Collection and Follow‐Up

Clinical and follow‐up data were retrospectively extracted from electronic medical records and institutional databases. Baseline clinicopathological variables included age, sex, smoking status, Eastern Cooperative Oncology Group performance status (ECOG‐PS), histological subtype, TNM stage, PD‐L1 expression level, treatment regimen, line of immunotherapy, genomic alterations, treatment response, immune‐related adverse events (irAEs), and survival status.

Molecular profiling data were obtained from clinically validated next‐generation sequencing (NGS) reports generated by certified commercial laboratories. Testing was performed on pretreatment tumor specimens as part of routine clinical care. As this was a real‐world study, different multigene NGS panels, typically covering several hundred cancer‐related genes, were used across patients. All samples met predefined quality‐control thresholds (tumor purity, DNA input, Q30 score, mean depth > 800×, mapping rate, and duplication rate). Variant calling applied a variant allele frequency (VAF) cutoff of ≥ 1% (or a platform‐specific validated threshold). Only somatic alterations consistently covered across the majority of panels and recognized as clinically relevant in NSCLC were included in the analyses. For survival analyses, TP53 status was treated as a binary variable (mutant versus wild type), without further subclassification.

Follow‐up information was obtained through routine outpatient visits, supplemented by telephone interviews. The data cut‐off date was October 31, 2024. Given the high proportion of patients with ECOG‐PS ≥ 2, ECOG‐PS was prespecified as a covariate in the multivariable models to account for its potential confounding effect on clinical outcomes.

### 2.3 Response Evaluation, Survival Outcomes, and Safety Assessment

Tumor response was assessed every 2–3 treatment cycles, or earlier if clinically indicated, using contrast‐enhanced CT or other appropriate imaging, according to the immune Response Evaluation Criteria in Solid Tumors (iRECIST). Assessments were not formally blinded; however, experienced radiologists reviewed all imaging using standardized iRECIST criteria to minimize subjective bias. Objective response rate (ORR) was defined as the proportion of patients achieving complete response (CR) or partial response (PR), and disease control rate (DCR) as the proportion achieving CR, PR, or stable disease (SD). Patients with initial unconfirmed progressive disease (iUPD) who remained clinically stable underwent repeat imaging to confirm progression or pseudoprogression, in line with iRECIST recommendations.

Progression‐free survival (PFS) was defined as the time from ICI initiation to documented progression or death from any cause, whichever occurred first. OS was defined as the time from ICI initiation to death from any cause. Patients without events were censored at the last follow‐up date. For equivocal radiological progression, treatment discontinuation or initiation of subsequent systemic therapy was considered as a PFS event.

irAEs were graded according to the Common Terminology Criteria for Adverse Events (CTCAE), version 5.0; the highest grade per patient was recorded.

### 2.4 PD‐L1 Expression Analysis

PD‐L1 expression was assessed prior to treatment initiation as part of routine clinical practice. Formalin‐fixed, paraffin‐embedded (FFPE) tumor specimens (diagnostic biopsy or surgical resection) were evaluated by immunohistochemistry using the PD‐L1 IHC 22C3 pharmDx assay (Agilent Technologies, Santa Clara, CA, USA), following the manufacturer's instructions. Testing was performed either at the study center or at certified external laboratories under standardized quality‐control procedures.

PD‐L1 expression was reported as the TPS, defined as the percentage of viable tumor cells showing partial or complete membranous staining of any intensity. PD‐L1‐high expression was defined as TPS ≥ 50%. For subgroup analyses, patients were further stratified into TPS 50%–89% and TPS ≥ 90% groups to explore potential biological and clinical heterogeneity within the PD‐L1‐high population [10, 11].

### 2.5 Pathological Evaluation of the Tumor Immune Microenvironment

Of the 91 patients, 75 had adequate, well‐preserved H&E‐stained sections with evaluable tumor‐stromal areas and were included in the immune microenvironment analysis. The remaining 16 cases were excluded due to poor tissue quality (e.g., section loss, severe fading, or tissue detachment).

TILs were evaluated on H&E sections according to the International Immuno‐Oncology Biomarkers Working Group guidelines [12]. Specimens were considered eligible only if they contained adequate evaluable stromal tissue and at least 100 viable tumor cells. Both biopsy and surgical specimens were included, with biopsies accounting for the majority of evaluable cases. Assessment was restricted to the stromal compartment within the invasive tumor area, excluding necrotic regions, crush artifacts, in situ components, and nonneoplastic tissue. To minimize sampling bias, pathologists reviewed the entire evaluable H&E section rather than selecting predefined high‐power fields or inflammatory hotspots. Stromal TIL density was estimated as the percentage of the total stromal area occupied by mononuclear inflammatory cells across all assessable invasive tumor regions. High TIL density was defined as ≥ 10%, and low density as < 10%.

TLSs were identified on H&E sections as lymphoid aggregates within the tumor bed or at the invasive margin and categorized as mature or immature according to the 2025 Chinese expert consensus [13]. Mature (secondary) TLSs were defined by the presence of a germinal center, appearing as a lighter central B‐cell zone surrounded by a darker T‐cell zone; immature (primary) TLSs consisted of dense lymphoid aggregates (≥ 50 cells) without a discernible germinal center. For survival analyses, TLSs were grouped as present (mature or immature) versus absent.

Two experienced pathologists independently assessed all cases in a blinded manner. Interobserver agreement, evaluated using Cohen's kappa coefficient, was 0.85 for TILs and 0.82 for TLSs. Discrepant cases were resolved by consensus or third‐pathologist review.

### 2.6 mIHC Analysis

To validate the biological basis of H&E‐based TIL stratification, mIHC was performed in a subset of 30 patients (15 with high TIL density, 15 with low TIL density) selected from the pathological evaluation cohort. FFPE tumor sections were stained using the PANO Multiplex IHC Kit (Panovue, Beijing, China).

Two antibody panels were applied: Panel A included CD68, HLA‐DR, CD163, PD‐L1, and Pan‐CK; Panel B included CD8, CD4, FOXP3, PD‐1, and Pan‐CK. Sequential immunostaining with tyramide signal amplification (TSA) was performed according to the manufacturer's protocol. Briefly, each target antigen was sequentially detected using primary and HRP‐conjugated secondary antibodies, followed by TSA‐based fluorescent signal deposition. Antibodies were stripped between cycles while retaining the fluorescent signals, enabling simultaneous detection of multiple markers on a single section.

Multispectral images were acquired using the Mantra quantitative pathology imaging system (PerkinElmer, USA) and analyzed with inForm software for spectral unmixing, autofluorescence subtraction, cell segmentation, and marker quantification. Immune cell densities were evaluated in tumor and stromal compartments. Analyzed parameters included stromal CD8+ T‐cell density, CD56(dim)+ and CD56(bright)+ natural killer cell density, and the M1/M2 macrophage ratio, calculated as the ratio of CD68 + HLA‐DR+ and CD68 + HLA‐DR− cells.

### 2.7 Statistical Analysis

Statistical analyses were performed using IBM SPSS Statistics (version 31.0) and GraphPad Prism (version 10.3.1). Continuous variables were compared with the independent‐samples *t*‐test or Mann–Whitney *U* test as appropriate, and categorical variables with the chi‐square test or Fisher's exact test. Survival curves were estimated by the Kaplan–Meier method and compared with the log‐rank test. Variables with *p* < 0.10 in univariable analysis were entered into multivariable Cox proportional hazards models. All tests were two‐sided; *p* < 0.05 was considered statistically significant.

## 3 Results

### 3.1 Patient Characteristics and Treatment Exposure

A total of 91 patients were included in the final analysis. The median follow‐up was 19.1 months. Baseline characteristics are summarized in Table 1. The median age was 67.2 years, with 67 patients (73.6%) aged ≥ 65 years; 70 (76.9%) were male. Never‐smokers accounted for 57 patients (62.6%). ECOG‐PS was 0–1 in 40 patients (44.0%) and ≥ 2 in 51 patients (56.0%). Most patients had stage IV disease (43 IVA, 27 IVB), while 21 (23.1%) had stage III. Adenocarcinoma was the predominant histological subtype (65/91, 71.4%), followed by squamous cell carcinoma (22/91, 24.2%). PD‐L1 TPS was 50%–89% in 68 patients (74.7%) and ≥ 90% in 23 (25.3%). Metastatic sites included bone (34, 37.4%), brain (17, 18.7%), and liver (10, 11.0%). Prior local treatment was infrequent: 18 patients (19.8%) had undergone surgery and 14 (15.4%) radiotherapy.

**TABLE 1:** Baseline clinical characteristics of patients.

Clinical characteristics	Number of patients	Percentage
Age		
< 65 years	24	26.4%
≥ 65 years	67	73.6%
Sex		
Male	70	76.9%
Female	21	23.1%
Smoking status		
Never	57	62.6%
Former or current	34	37.4%
ECOG‐PS		
0–1	40	44.0%
≥ 2	51	56.0%
TNM stage		
III	21	23.1%
IVA	43	47.3%
IVB	27	29.7%
Histological subtype		
Adenocarcinoma	65	71.4%
Squamous	22	24.2%
Others	4	4.4%
PD‐L1 status		
TPS 50%–89%	68	74.7%
TPS 90%–100%	23	25.3%
Metastasis		
Bone	34	37.4%
Brain	17	18.7%
Liver	10	11.0%
Treatment regimen		
Immunotherapy alone	15	16.5%
Immunotherapy plus chemotherapy	44	48.4%
Immunotherapy plus targeted therapy	8	8.8%
Immunotherapy plus chemotherapy and targeted therapy	24	26.3%
Line of immunotherapy		
First‐line	70	76.9%
Second‐line	9	9.9%
≥ Third‐line	12	13.2%
irAEs		
Grade 1	57	62.6%
Grade 2	23	25.3%
Grade 3	9	9.9%
Grade 4	2	2.2%
Grade 5	0	0.0%

Abbreviations: ECOG‐PS, Eastern Cooperative Oncology Group Performance Status; irAEs, immune‐related adverse events; PD‐1, programmed cell death protein‐1; TNM, tumor–node–metastasis; TPS, tumor proportion score.

Overall, 70 patients (76.9%) received ICIs as first‐line therapy. Combination therapy with chemotherapy was the most common regimen (44/91, 48.4%), followed by ICIs combined with chemotherapy and targeted therapy (24, 26.3%); ICI monotherapy was administered in 15 patients (16.5%). Sintilimab was the most frequently used agent (41/91, 45.1%), followed by pembrolizumab (23, 25.3%), tislelizumab (14, 15.4%), serplulimab (9, 9.9%), and camrelizumab (4, 4.4%). IrAEs were mostly Grades 1–2 (80/91, 87.9%); Grade 3 and Grade 4 irAEs occurred in 9 (9.9%) and 2 (2.2%) patients, respectively. No Grade 5 irAEs were recorded. In summary, this was a predominantly elderly cohort with advanced‐stage disease, most of whom received first‐line ICI‐based combination therapy.

### 3.2 Genomic Landscape and Association With PD‐L1 Expression

**FIGURE 1:**
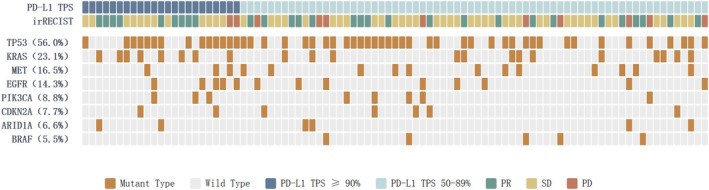
Genomic landscape and best overall response in patients with PD‐L1‐high advanced NSCLC. Oncoprint showing the distribution of recurrent somatic alterations and best overall response in the 91 included patients. Each column represents one patient. The upper annotations indicate PD‐L1 subgroup (TPS 50%–89% or TPS 90%–100%) and best radiographic response. The most frequently altered genes were TP53 (56.0%), KRAS (23.1%), MET (16.5%), and EGFR (14.3%), followed by PIK3CA, CDKN2A, ARID1A, and BRAF.

To characterize the molecular features of this cohort, next‐generation sequencing (NGS) data were analyzed. Somatic alterations were identified in 72 of 91 patients (79.1%). The most frequently altered genes were TP53 (51/91, 56.0%), KRAS (21, 23.1%), MET (15, 16.5%), and EGFR (13, 14.3%), followed by PIK3CA (8, 8.8%), CDKN2A (7, 7.7%), ARID1A (6, 6.6%), and BRAF (5, 5.5%). The overall genomic landscape is presented in Figure 1. Co‐occurring alterations were frequently observed; notably KRAS/TP53 co‐mutation identified in 12 patients (57.1% of all KRAS‐mutant cases).

The ORR was 42.9% (9/21) in KRAS‐mutant tumors, 27.5% (14/51) in TP53‐mutant tumors, and 20.0% (3/15) in MET‐altered tumors. EGFR‐mutant tumors exhibited the lowest ORR (7.7%, 1/13) and the highest rate of progressive disease (PD) (46.2%, 6/13). Among the 13 patients with EGFR‐mutant tumors, seven received first‐line ICIs without prior EGFR‐TKI exposure, whereas six received ICIs after progression on EGFR‐TKI‐based therapy.

No significant association was observed between any major genomic alterations and very high PD‐L1 expression (TPS ≥ 90%) (Table 2), including TP53 (72.5% vs. 77.5%, *p* = 0.590), KRAS (61.9% vs. 78.6%, *p* = 0.123), MET (80.0% vs. 73.7%, *p* = 0.607), and EGFR (61.5% vs. 76.9%, *p* = 0.237). Overall, recurrent genomic alterations were frequently detected in this cohort but were not significantly associated with PD‐L1 expression.

**TABLE 2:** Association between gene mutation status and PD‐L1 status.

Gene mutation status	Number of patients	PD‐L1 TPS ≥ 90% [*n* (%)]	*χ* ^2^	*p*
TP53				
Mutant	51	37 (72.5)	0.291	0.590
Wild‐type	40	31 (77.5)		
KRAS				
Mutant	21	13 (61.9)	2.376	0.123
Wild‐type	70	55 (78.6)		
MET				
Mutant	15	12 (80.0)	0.265	0.607
Wild‐type	76	56 (73.7)		
EGFR				
Mutant	13	8 (61.5)	1.396	0.237
Wild‐type	78	60 (76.9)		

*Note:* All *p* values were > 0.05, indicating no statistically significant differences.

Abbreviations: EGFR, epidermal growth factor receptor; KRAS, Kirsten rat sarcoma viral oncogene homolog; MET, mesenchymal–epithelial transition factor; PD‐1, programmed cell death protein‐1; TP53, tumor protein p53; TPS, tumor proportion score.

### 3.3 Treatment Response and Subgroup Analyses

Based on the above clinical and molecular characteristics, we evaluated the efficacy of ICI‐based therapy in all 91 patients. No CRs were observed. PR was achieved in 29 patients (31.9%), SD in 51 (56.0%), and PD in 11 (12.1%), yielding an ORR of 31.9% and a DCR of 87.9%. Patients with TPS ≥ 90% had a numerically higher ORR than those with TPS 50%–89% (39.1% vs. 29.4%, *p* = 0.442) (Table 3). Two patients (2.2%) initially met iUPD criteria and subsequently achieved PR following continued ICI treatment, consistent with pseudoprogression.

**TABLE 3:** Subgroup analysis of treatment efficacy according to key clinical characteristics.

Clinical characteristics	Number of patients	PR + CR	ORR (95% CI, %)	*p*
Overall cohort	91	29	31.87	
PD‐L1 status				
TPS 50%–89%	68	20	29.41 (18.98–41.71)	0.442
TPS 90%–100%	23	9	39.13 (19.71–61.46)	
ECOG‐PS				
0–1	40	18	45.00 (29.26–61.51)	**0.024** ^a^
≥ 2	51	11	21.57 (11.29–35.32)	
Line of immunotherapy				
First‐line	70	27	38.57 (27.23–50.94)	**0.016** ^a^
≥ Second‐line	21	2	9.52 (1.17–30.38)	
EGFR				
Mutant	13	1	7.69 (0.19–36.03)	**0.046** ^a^
Wild‐type	78	28	35.90 (25.30–47.62)	
Mutation sites				0.066
0	19	7	36.84 (16.29–61.64)	
1	43	9	20.93 (10.02–36.05)	**0.043** ^a^
≥ 2	29	13	44.83 (26.45–64.30)	

*Note:* The “number of gene mutation sites” included only somatic driver mutations with established clinical significance. For the comparison among the three groups stratified by number of gene mutation sites, the overall *p* value was 0.066; however, the comparison between the 1 mutation site group and the ≥ 2 mutation sites group was statistically significant (*p* = 0.043).

Abbreviations: CR, complete response; ECOG‐PS, Eastern Cooperative Oncology Group Performance Status; EGFR, epidermal growth factor receptor; ORR, objective response rate; PD‐1, programmed cell death protein‐1; PR, partial response; TPS, tumor proportion score.

^a^ The bold values in denote statistically significant results (*P* < 0.05).

In subgroup analyses, patients with ECOG‐PS 0–1 had a higher ORR than those with ECOG‐PS ≥ 2 (45.0% vs. 21.6%, *p* = 0.024). First‐line ICI treatment was also associated with a higher ORR than later‐line treatment (38.6% vs. 9.5%, *p* = 0.016). At the molecular level, patients harboring EGFR‐mutant tumors had a lower ORR than those with EGFR wild‐type tumors (7.7% vs. 35.9%, *p* = 0.046). Although the overall comparison among mutation‐number subgroups did not reach statistical significance (*p* = 0.066), patients with ≥ 2 clinically relevant genomic alterations exhibited a higher ORR than those with a single alteration (44.8% vs. 20.9%, *p* = 0.043).

These findings suggest that treatment response was more strongly associated with clinical status and specific molecular features than with the degree of PD‐L1 expression within the PD‐L1‐high population.

### 3.4 Survival According to PD‐L1 Subgroup and Genomic Features

**FIGURE 2:**
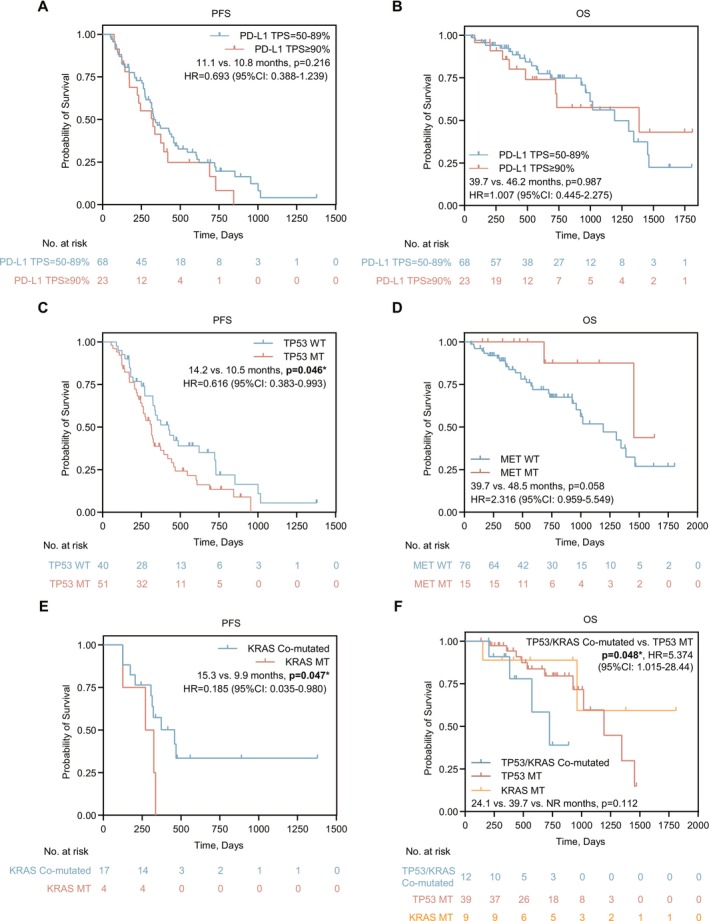
Kaplan–Meier analyses of survival according to PD‐L1 subgroup and key molecular features. (A) Progression‐free survival (PFS) according to PD‐L1 subgroup (TPS 50%–89% vs. TPS ≥ 90%). (B) Overall survival (OS) according to PD‐L1 subgroup. (C) PFS according to TP53 mutation status (wild‐type vs. mutant). (D) OS according to MET alteration status (wild‐type vs. mutant). (E) PFS according to KRAS mutation pattern (KRAS co‐mutated vs. KRAS single mutation). (F) OS according to co‐mutation pattern among patients with TP53/KRAS co‐mutation, TP53 single mutation, and KRAS single mutation. MT, mutant; OS, overall survival; PFS, progression‐free survival; WT, wild type. *p < 0.05.

To determine whether the observed differences in treatment response translated into differences in survival, survival outcomes were analyzed. The median PFS was 10.7 months, and the median OS was 43.5 months. No significant survival differences were observed between PD‐L1 subgroups (TPS 50%–89% vs. TPS ≥ 90%: median PFS 11.1 vs. 10.8 months, *p* = 0.216; median OS 39.7 vs. 46.2 months, *p* = 0.987) (Figure 2A,B), suggesting that further PD‐L1 stratification beyond TPS ≥ 50% provides limited additional prognostic value.

Among individual genomic alterations, TP53‐mutant patients had a shorter median PFS than TP53 wild‐type patients (10.5 vs. 14.2 months, *p* = 0.046; Figure 2C), with no significant OS difference. KRAS, MET, and EGFR alterations were not significantly associated with survival, although MET alteration showed a trend toward improved OS (48.5 vs. 39.7 months, *p* = 0.058; Figure 2D). In co‐mutation analyses, patients with KRAS co‐mutations had longer PFS than those with isolated KRAS mutations (15.3 vs. 9.9 months, HR = 0.19, *p* = 0.047; Figure 2E), whereas TP53/KRAS co‐mutation was associated with shorter OS compared with TP53 mutation alone (24.1 vs. 39.7 months, HR = 5.37, *p* = 0.048; Figure 2F). Additional exploratory survival analyses are shown in Figures S1 and S2.

Collectively, these results indicate that PD‐L1 expression level alone did not meaningfully stratify survival in PD‐L1‐high NSCLC, whereas specific genomic alterations and co‐mutation patterns were associated with distinct survival outcomes following ICI‐based therapy.

### 3.5 Survival According to Tumor Immune Microenvironment Features and Joint Stratification With PD‐L1 Expression

**FIGURE 3:**
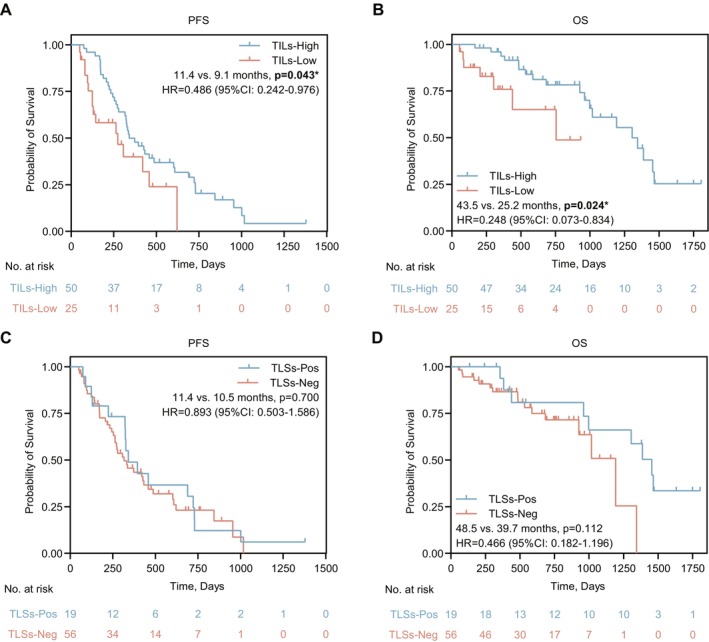
Kaplan–Meier analyses of survival according to tumor immune microenvironment features. (A) Progression‐free survival (PFS) according to tumor‐infiltrating lymphocyte (TIL) density (TILs‐High vs. TILs‐Low). (B) Overall survival (OS) according to TIL density. (C) PFS according to tertiary lymphoid structure (TLS) status (TLSs‐Pos vs. TLSs‐Neg). (D) OS according to TLS status. Neg, negative; OS, overall survival; PFS, progression‐free survival; Pos, positive; TILs, tumor‐infiltrating lymphocytes; TLSs, tertiary lymphoid structures. *p < 0.05.

**FIGURE 4:**
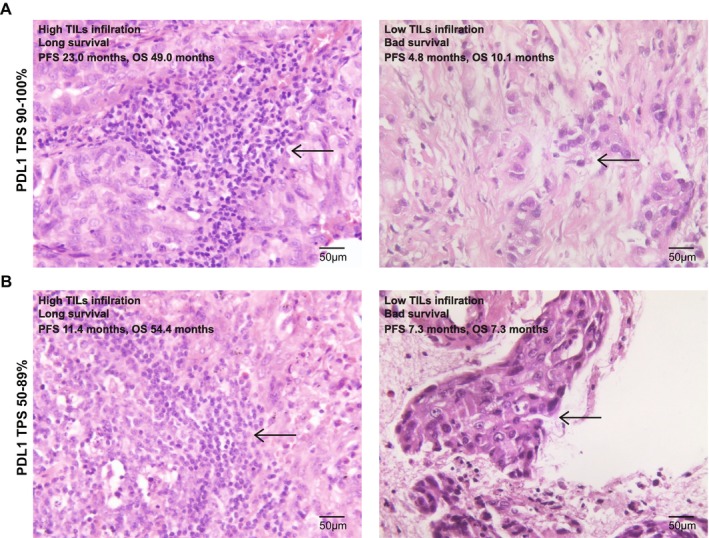
Representative histology according to PD‐L1 expression level and tumor‐infiltrating lymphocyte (TIL) density. (A) Representative histological sections (H&E staining) from two patients with PD‐L1 TPS = 90%–100%. (B) Representative histological sections from two patients with PD‐L1 TPS = 50%–89%. All histological images were captured at ×400 magnification; scale bars represent 50 μm. OS, overall survival; PD‐L1, programmed death‐ligand 1; PFS, progression‐free survival; TILs, tumor‐infiltrating lymphocytes; TPS, tumor proportion score.

Because genetic alterations may shape the tumor immune microenvironment, which is a critical determinant of ICI efficacy, we further examined immune‐related pathological features in 75 patients with available pretreatment H&E‐stained sections and assessed their association with survival outcomes. Survival analysis showed that high TIL density was associated with longer both PFS (11.4 vs. 9.1 months, *p* = 0.043; Figure 3A) and OS (43.5 vs. 25.2 months, *p* = 0.024; Figure 3B). In contrast, TLS presence was not significantly associated with either PFS (11.4 vs. 10.5 months, *p* = 0.700; Figure 3C) or OS (48.5 vs. 39.7 months, *p* = 0.112; Figure 3D). Representative H&E images illustrating differential TIL density and corresponding clinical outcomes are shown in Figure 4.

**FIGURE 5:**
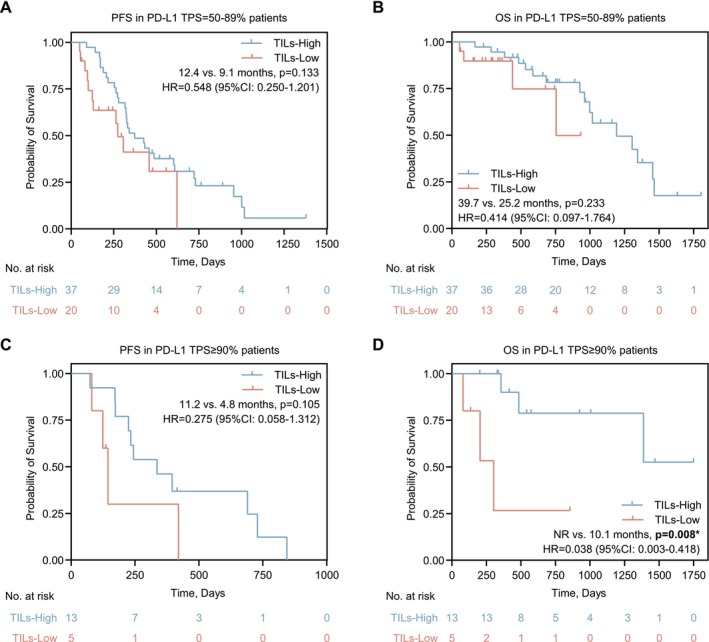
Joint stratification of survival by PD‐L1 expression level and tumor‐infiltrating lymphocyte (TIL) density. (A) Progression‐free survival (PFS) in the PD‐L1 TPS 50%–89% subgroup stratified by TIL density (TILs‐High vs. TILs‐Low). (B) Overall survival (OS) in the PD‐L1 TPS 50%–89% subgroup according to TIL density. (C) PFS in the PD‐L1 TPS ≥ 90% subgroup stratified by TIL density. (D) OS in the PD‐L1 TPS ≥ 90% subgroup according to TIL density. NR, not reached; OS, overall survival; PD‐L1, programmed death‐ligand 1; PFS, progression‐free survival; TILs, tumor‐infiltrating lymphocytes; TPS, tumor proportion score. *p < 0.05.

We next performed joint stratification analyses to evaluate the combined effect of PD‐L1 expression and TIL density. In the TPS 50%–89% subgroup, high TIL density was associated with numerically longer PFS (12.4 vs. 9.1 months) and OS (39.7 vs. 25.2 months), although neither difference reached statistical significance (Figure 5A,B). In contrast, among patients with TPS ≥ 90%, high TIL density was associated with significantly prolonged OS (median not reached vs. 10.1 months, *p* = 0.008), whereas no significant difference in PFS (11.2 vs. 4.8 months, *p* = 0.105; Figure 5C,D).

Together, these findings support the prognostic value of TIL density and suggest that incorporating tumor immune microenvironment may further refine prognostic stratification beyond PD‐L1 expression alone, particularly among patients with TPS ≥ 90%.

### 3.6 Multiplex Immunohistochemical Profiling of TIL‐Associated Immune Phenotypes

**FIGURE 6:**
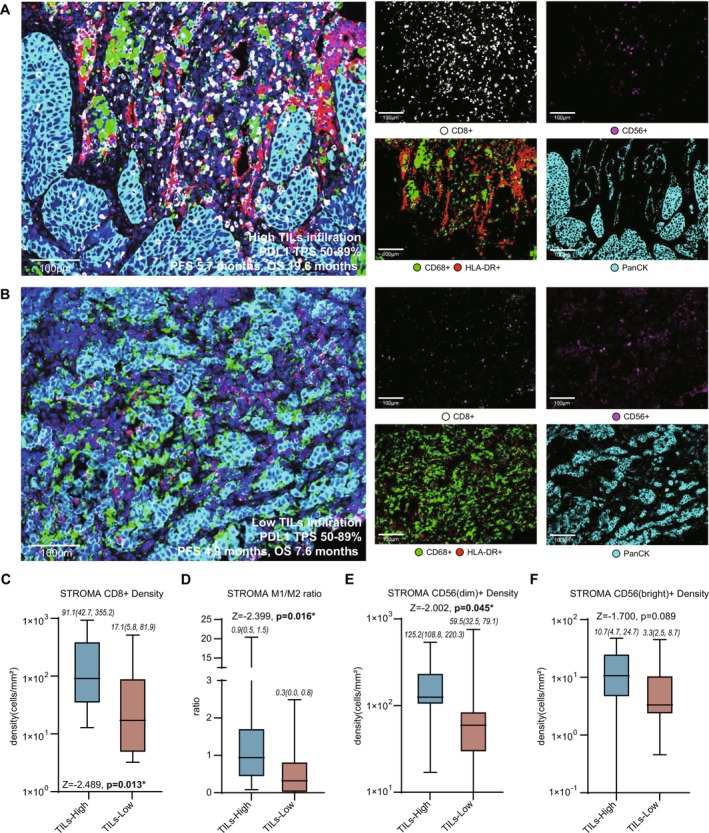
Multiplex immunohistochemical profiling of TIL‐associated immune phenotypes. (A and B) Representative multiplex immunohistochemistry (mIHC) images evaluating the immune cellular composition in pretreatment tumor tissues classified as TILs‐High (A) and TILs‐Low (B). (C) Stromal CD8+ T‐cell density in the TILs‐High group compared to the TILs‐Low group. (D) Stromal M1/M2 macrophage ratio in TILs‐High versus TILs‐Low tumors. (E) Density of CD56(dim) + natural killer (NK) cells in the TILs‐High and TILs‐Low groups. (F) Density of CD56(bright) + NK‐cells in the TILs‐High and TILs‐Low groups. CD, cluster of differentiation; HLA‐DR, human leukocyte antigen‐DR; mIHC, multiplex immunohistochemistry; NK, natural killer; PanCK, pan‐cytokeratin; TILs, tumor‐infiltrating lymphocytes. *p < 0.05.

To validate the immune cellular composition underlying TIL‐based stratification, mIHC was performed in 30 patients with available pretreatment tumor tissues, classified as TILs‐High (*n* = 15) and TILs‐Low (*n* = 15) according to H&E‐based pathological assessment. Representative mIHC images are shown in Figure 6A,B.

High‐TIL tumors exhibited a more immune‐enriched phenotype characterized by increased infiltration of multiple immune cell populations. Stromal CD8+ T‐cell density was higher in the TILs‐High group compared than in the TILs‐Low group (median [IQR]: 91.10 [42.74–355.21] vs. 17.10 [5.78–81.90] cells/mm^2^, *p* = 0.013; Figure 6C). Stromal M1/M2 macrophage ratio was also elevated in TILs‐High tumors (0.94 [0.50–1.55] vs. 0.32 [0.04–0.80], *p* = 0.016; Figure 6D). Consistent with enhanced immune activation, TILs‐High tumors demonstrated higher densities of CD56(dim) + natural killer (NK) cells (125.20 [108.75–220.33] vs. 59.51 [32.48–79.10] cells/mm^2^, *p* = 0.008; Figure 6E). CD56(bright) + NK‐cell density was numerically higher in the high‐TIL group, but the difference did not reach statistical significance (10.67 [4.73–24.69] vs. 3.32 [2.46–8.71] cells/mm^2^, *p* = 0.089; Figure 6F).

Collectively, these findings provide biological validation that H&E‐based TIL assessment captures a genuinely immune‐inflamed tumor microenvironment characterized by coordinated enrichment of cytotoxic T cells, NK‐cell subsets, and a proinflammatory macrophage polarization profile.

### 3.7 Independent Prognostic Factors for PFS and OS

Finally, variables with *p* < 0.10 in univariable analyses were entered into multivariable Cox regression models to identify independent prognostic factors for PFS and OS (Table 4). For PFS, age ≥ 65 years (HR = 3.07, 95% CI: 1.48–6.36, *p* = 0.003) and TP53 mutation (HR = 1.80, 95% CI: 1.09–3.53, *p* = 0.027) were independently associated with shorter PFS, whereas high TIL density was independently protective (HR = 0.65, 95% CI: 0.36–0.94, *p* = 0.035). Grade 4 irAEs were also associated with shorter PFS (HR = 6.05, 95% CI: 1.24–29.51, *p* = 0.026); however, this association should be interpreted cautiously given that only two patients experienced Grade 4 events. For OS, ECOG‐PS ≥ 2 (HR = 3.13, 95% CI: 1.21–8.10, *p* = 0.019), stage IVB disease (HR = 2.29, 95% CI: 1.09–4.79, *p* = 0.031), and bone metastasis (HR = 2.84, 95% CI: 1.03–7.84, *p* = 0.044) were independent adverse prognostic factors, while high TIL density remain independently associated with improved OS (HR = 0.53, 95% CI: 0.18–0.95, *p* = 0.044).

**TABLE 4:** Univariate and multivariate Cox regression model for predicting PFS and OS.

	PFS, univariable		PFS, multivariable		OS, univariable		OS, multivariable	
	HR (95% CI)	*P*	HR (95% CI)	*p*	HR (95% CI)	*p*	HR (95% CI)	*p*
PD‐L1 status								
TPS 50%–89%	Reference				Reference			
TPS 90%–100%	1.398 (0.820–2.386)	0.219			0.735 (0.281–1.925)	0.531		
Age								
	Reference		Reference		Reference			
≥ 65 years	2.640 (1.440–4.840)	**0.002** ^a^	3.065 (1.476–6.363)	**0.003** ^a^	1.535 (0.684–3.443)	0.299		
ECOG‐PS								
0–1	Reference		Reference		Reference		Reference	
≥ 2	1.555 (0.966–2.503)	0.069	1.363 (0.800–2.320)	0.254	3.056 (1.368–6.826)	**0.006** ^a^	3.127 (1.207–8.097)	**0.019** ^a^
TNM stage								
III	Reference				Reference		Reference	
IVA	0.971 (0.516–1.830)	0.929			1.970 (0.566–6.861)	0.287	1.686 (0.469–6.066)	0.424
IVB	1.626 (0.840–3.144)	0.149			3.576 (1.016–12.58)	**0.047** ^a^	2.285 (1.091–4.785)	**0.031** ^a^
Bone metastasis								
No	Reference				Reference		Reference	
Yes	1.339 (0.835–2.149)	0.266			2.418 (1.184–4.941)	**0.015** ^a^	2.839 (1.028–7.835)	**0.044** ^a^
irAEs								
Grade 1	Reference		Reference		Reference			
Grade 2	0.987 (0.568–1.715)	0.964	1.139 (0.628–2.064)	0.668	1.238 (0.561–2.732)	0.598		
Grade 3	1.110 (0.517–2.383)	0.789	1.230 (0.493–3.071)	0.657	0.877 (0.255–3.018)	0.836		
Grade 4	4.257 (1.001–18.10)	0.050	6.046 (1.239–29.51)	**0.026** ^a^	2.957 (0.384–22.74)	0.298		
TP53								
Wild‐type	Reference		Reference		Reference			
Mutant	1.631 (1.002–2.657)	**0.049** ^a^	1.798 (1.087–3.526)	**0.027** ^a^	1.070 (0.508–2.256)	0.858		
TILs density								
Low	Reference		Reference		Reference		Reference	
High	0.540 (0.294–0.990)	**0.046** ^a^	0.648 (0.359–0.940)	**0.035** ^a^	0.335 (0.124–0.906)	**0.031** ^a^	0.526 (0.178–0.951)	**0.044** ^a^

Abbreviations: ECOG‐PS, Eastern Cooperative Oncology Group Performance Status; irAEs, immune‐related adverse events; PD‐1, programmed cell death protein‐1; TILs, tumor‐infiltrating lymphocytes; TNM, tumor–node–metastasis; TP53, tumor protein p53; TPS, tumor proportion score.

^a^ The bold values in denote statistically significant results (*P* < 0.05).

Overall, these findings indicate that molecular alterations, immune microenvironment features, and established clinical factors independently contribute to survival heterogeneity in PD‐L1‐high advanced NSCLC treated with ICI‐based therapy.

## 4 Discussion

In this retrospective cohort of 91 patients with PD‐L1‐high advanced NSCLC receiving ICI‐based therapy, we observed substantial outcome heterogeneity that was not explained by PD‐L1 expression alone. Integrating genomic profiling with pathological assessment of the tumor immune microenvironment revealed that TP53 mutation and TIL density contributed to this variability. Overall efficacy was clinically meaningful, with an ORR of 31.9%, a DCR of 87.9%, a median PFS of 10.7 months, and a median OS of 43.5 months, outcomes broadly consistent with pivotal clinical trials of immunotherapy in PD‐L1‐high NSCLC [7, 14], supporting the real‐world generalizability of our findings.

A key finding was that PD‐L1 expression alone did not further discriminate response or survival once the TPS ≥ 50% threshold was reached. Patients with TPS 50%–89% and those with TPS ≥ 90% exhibited comparable ORR (29.4% vs. 39.1%, *p* = 0.442), median PFS (11.1 vs. 10.8 months, *p* = 0.216), and median OS (39.7 vs. 46.2 months, *p* = 0.987). Thus, while PD‐L1 remains essential for selecting patients for immunotherapy, higher expression within the PD‐L1‐high subgroup provides limited additional prognostic value, consistent with evidence that the discriminatory power of PD‐L1 diminishes once patients are enriched for benefit [10, 11]. Biologically, PD‐L1 upregulation reflects adaptive immune resistance, oncogenic signaling, and microenvironmental regulation—rather than a simple linear indicator of antitumor immunity [8, 9]. In keeping with this, TP53, KRAS, MET, and EGFR alterations were not significantly associated with the TPS ≥ 90% subgroup in our cohort.

Among the molecular features, TP53 mutation emerged as the most relevant adverse prognostic factor. It was the most frequent alteration (56.0%, 51/91) and independently predicted shorter PFS (HR = 1.80, *p* = 0.027). Patients with TP53‐mutant tumors also had a shorter median PFS than wild‐type (10.5 vs. 14.2 months, *p* = 0.046), but no significant OS difference, suggesting a greater impact on early disease control—an endpoint more susceptible to subsequent treatment influences. Previous studies on TP53 as an ICI biomarker have yielded inconsistent results [15, 16]. Our data support that TP53 mutation may identify a subgroup with more aggressive tumor biology and less durable immunotherapy benefit in PD‐L1‐high NSCLC. Discrepancies may partly stem from the biological heterogeneity of TP53 alterations; here, TP53 was analyzed as a binary variable without distinguishing missense, nonsense, or truncating mutations, which can differentially affect p53 function and immune interactions [17]. Thus, our findings reflect the overall impact of TP53 alterations rather than specific subtypes. Future studies incorporating functional annotation of TP53 mutations are needed to refine their prognostic and predictive significance.

Co‐mutation analyses further suggest that genomic context may outperform single‐gene status. KRAS co‐mutations were associated with longer PFS than isolated KRAS mutation (15.3 vs. 9.9 months, HR = 0.19, *p* = 0.047), whereas TP53/KRAS co‐mutation predicted shorter OS than TP53 mutation alone (24.1 vs. 39.7 months, HR = 5.37, *p* = 0.048). Given the limited sample size and borderline significance, these observations remain hypothesis‐generating but align with evidence that KRAS‐mutant NSCLC is heterogeneous and that co‐occurring TP53 alterations profoundly influence immune phenotype and clinical outcomes [18, 19, 20].

Another notable finding was the poor response of EGFR‐mutant tumors. Despite high PD‐L1 expression, EGFR‐mutant patients had the lowest ORR (7.7%, 1/13) and the highest PD rate (46.2%, 6/13), consistent with an immune‐cold microenvironment marked by reduced effector T‐cell infiltration [21]. Importantly, treatment exposure was heterogeneous: seven patients received first‐line ICIs without prior EGFR‐TKI exposure, while six received ICIs post‐TKI progression. Hence, the lack of survival difference should be interpreted cautiously, reflecting small numbers, varied treatment sequences, and the influence of subsequent targeted therapy [22].

A strength of this study is the integration of pathological immune microenvironment features into prognostic assessment. Among 75 evaluable patients, high TIL density was associated with longer PFS (11.4 vs. 9.1 months, *p* = 0.043) and OS (43.5 vs. 25.2 months, *p* = 0.024), and remained an independently protective factor for both PFS (HR = 0.65) and OS (HR = 0.53). In contrast, TLSs were not significantly associated with survival, which may reflect the limited sample size and the inherent complexity of TLS maturation and spatial organization [23]. The prognostic value of TIL density was further supported by mIHC analysis in an exploratory subset of 30 patients. Compared with TIL‐low tumors, TIL‐high tumors exhibited an immune‐enriched phenotype characterized by increased stromal CD8+ T‐cell infiltration, higher CD56(dim) + NK‐cell density, and a more proinflammatory M1/M2 macrophage profile. These findings suggest that routine H&E‐based TIL assessment captures broader immune contexture rather than merely lymphocyte abundance.

The joint analysis of PD‐L1 expression and TIL density provided additional biological insight [24]. Among patients with TPS ≥ 90%, high TIL density identified a subgroup with markedly prolonged OS (not reached vs. 10.1 months, *p* = 0.008), whereas this association was less evident in the TPS 50%–89% subgroup. This finding indicates that very high PD‐L1 expression is biologically heterogeneous and does not uniformly represent an immune‐active tumor state. When accompanied by robust T‐cell infiltration, high PD‐L1 expression likely reflect adaptive immune resistance driven by interferon‐γ‐mediated PD‐L1 induction [8, 25], indicating ongoing dependence on the PD‐1/PD‐L1 pathway. Conversely, in the absence of substantial T‐cell infiltration, very high PD‐L1 expression may represent an immune‐excluded phenotype or tumor‐intrinsic oncogenic PD‐L1 upregulation rather than adaptive immune resistance [9, 26]. In these tumors, high TPS may signify immune escape without effective antitumor immune activation, potentially explaining the inferior survival observed in the TPS ≥ 90%/TIL‐low subgroup. Collectively, these findings suggest that TIL density provides complementary biological information beyond PD‐L1 expression alone and may help distinguish immune‐active and immune‐excluded subsets within PD‐L1‐high NSCLC.

In addition to molecular and immune variables, host‐related clinical factors remained important determinants of outcome. ECOG‐PS ≥ 2 was independently associated with worse OS (HR = 3.13, *p* = 0.019) and a lower ORR (21.6% vs. 45.0%, *p* = 0.024), underscoring the prognostic importance of baseline functional status [27]. Notably, 56.0% of our patients had ECOG‐PS ≥ 2, a proportion exceeding that in pivotal trials, reflecting real‐world practice. Although this imbalance may have influenced observed efficacy, ECOG‐PS ≥ 2 remained an independent adverse prognostic factor after multivariable adjustment; therefore, performance status should be considered when generalizing our findings. Age ≥ 65 years independently predicted shorter PFS (HR = 3.07, *p* = 0.003), possibly reflecting immunosenescence [28]. Stage IVB disease (HR = 2.29, *p* = 0.031) and bone metastasis (HR = 2.84, *p* = 0.044) were independently associated with inferior OS, consistent with evidence that bone involvement portends poorer outcomes in ICI‐treated NSCLC [29, 30]. Grade 4 irAEs were independently associated with shorter PFS (HR = 6.05, *p* = 0.026), but this estimate is highly unstable because only two patients experienced Grade 4 events; severe irAEs can necessitate treatment interruption and immunosuppressive therapy, potentially compromising benefit [31].

Several limitations should be acknowledged. This retrospective single‐center study had a modest sample size, limiting statistical power for subgroup and co‐mutation analyses and for evaluating rare events such as Grade 4 irAEs. Despite multivariable adjustment, residual confounding from treatment heterogeneity (different ICI agents, regimens, and lines) cannot be excluded. TIL and TLS assessments were based on H&E‐stained sections rather than quantitative multiplex immune profiling, and TP53 mutations were analyzed as a binary variable without functional subclassification; both factors may have reduced the granularity of our immunogenomic characterization. The use of multiple commercially NGS platforms may have introduced inter‐patient variability in mutation detection. Additionally, the high proportion of patients with ECOG‐PS ≥ 2, while reflective of real‐world practice, may limit the generalizability to fitter populations typical of pivotal trials. Importantly, the combined genomic and immune markers presented here should not be regarded as hypothesis‐generating; their clinical utility requires prospective validation in large‐scale multicenter cohorts.

## 5 Conclusion

This study demonstrates that genomic and immune microenvironment features can refine prognostic stratification beyond PD‐L1 expression alone in PD‐L1‐high advanced NSCLC. TP53 mutation was associated with shorter PFS, whereas high TIL density—particularly within the TPS ≥ 90% subgroup—predicted more favorable survival. mIHC confirmed that high‐TIL tumors harbor an immune‐enriched microenvironment, providing biological support for TIL‐based stratification. However, given the retrospective, single‐center design and limited sample size, these findings are hypothesis‐generating and require validation in large‐scale prospective multicenter cohorts incorporating multiplex immune profiling and standardized genomic platforms.

## Author Contributions

**Hui Yan:** conceptualization, methodology, data curation, investigation, validation, formal analysis, visualization, writing – original draft, writing – review and editing. **Qian Xu:** writing – original draft, writing – review and editing, data curation, investigation, validation. **Yushuang Zheng:** methodology, data curation, investigation, validation, writing – review and editing. **Dapeng Li:** conceptualization, writing – review and editing, resources, project administration, methodology, funding acquisition. **Meng Shen:** writing – review and editing, resources, investigation, supervision, visualization.

## Funding

This work was supported by Suzhou Basic Research Pilot Project (SSD2024059).

## Ethics Statement

The study was conducted in accordance with the Declaration of Helsinki and its subsequent amendments. The retrospective analysis was conducted under the ethics approval of the First Affiliated Hospital of Soochow University (No. 2025347) and written informed consent was obtained from all participants prior to their inclusion in the study.

## Consent

Patients provided written informed consent for the publication of any data and accompanying images.

## Conflicts of Interest

The authors declare no conflicts of interest.

## Supporting information


**Figure S1:** Kaplan–Meier analyses of survival according to gene mutation status in PD‐L1 high‐expression NSCLC patients.

**Figure S2:** Kaplan–Meier analyses of survival according to co‐mutation status in PD‐L1 high‐expression NSCLC patients.

## Data Availability

The data that support the findings of this study are available from the corresponding author upon reasonable request.
