# Supplementary material for: TP53 Mutation and Tumor‐Infiltrating Lymphocytes as Predictors of PD‐1/PD‐L1 Inhibitor Efficacy in PD‐L1‐High Advanced NSCLC
Source: Cancer Rep (Hoboken). 2026 Aug 13;9(8):e70648. doi: 10.1002/cnr2.70648 (PMC13473177; doi:10.1002/cnr2.70648)
Supplement: Supplementary file 1 [file CNR2-9-e70648-s001.pdf]

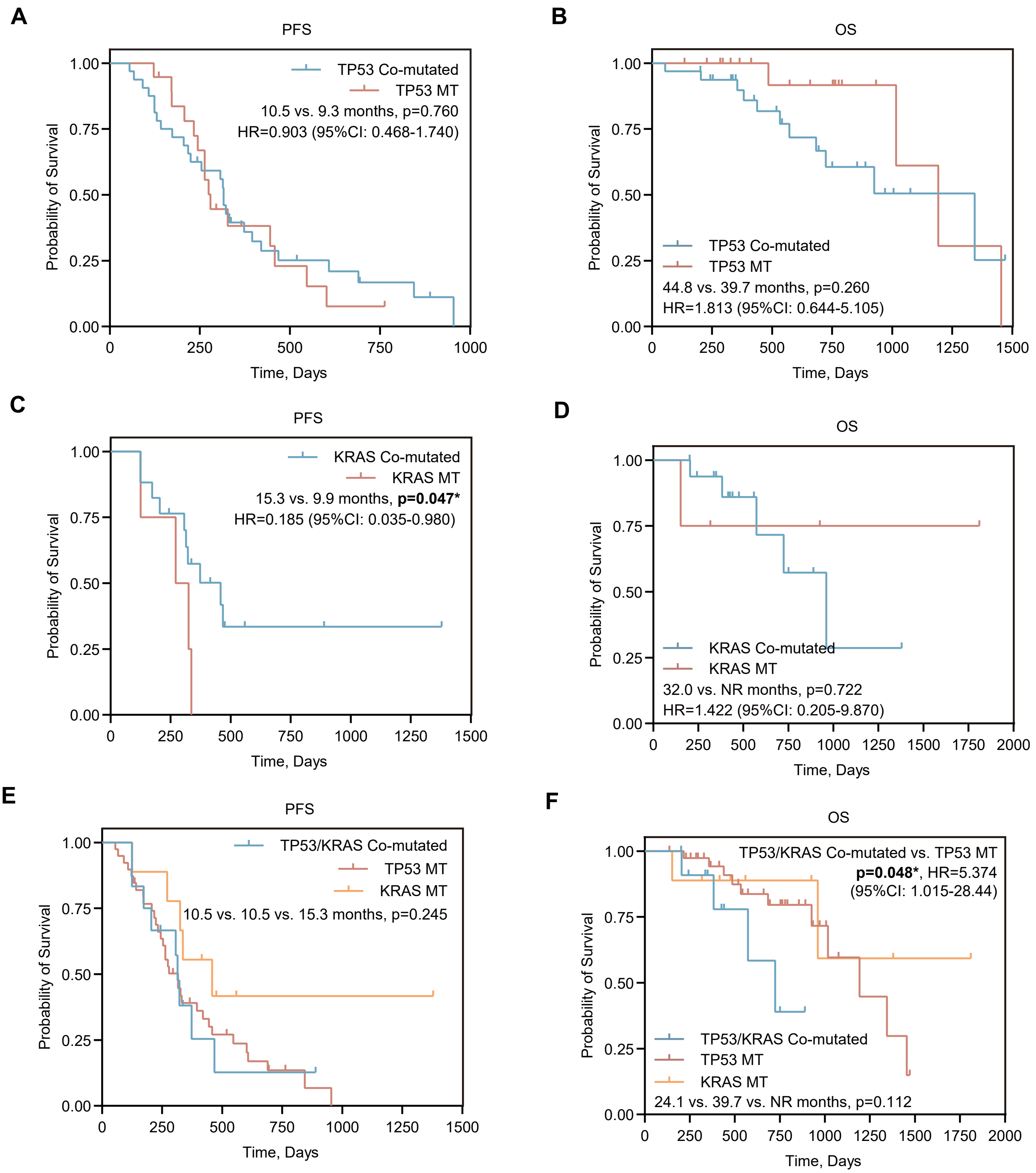
A
PFS
TP53 Co-mutated
TP53 MT
10.5 vs. 9.3 months, p=0.760
HR=0.903 (95%CI: 0.468-1.740)
Probability of Survival
Time, Days
B
OS
TP53 Co-mutated
TP53 MT
44.8 vs. 39.7 months, p=0.260
HR=1.813 (95%CI: 0.644-5.105)
Probability of Survival
Time, Days
C
PFS
KRAS Co-mutated
KRAS MT
15.3 vs. 9.9 months, p=0.047*
HR=0.185 (95%CI: 0.035-0.980)
Probability of Survival
Time, Days
D
OS
KRAS Co-mutated
KRAS MT
32.0 vs. NR months, p=0.722
HR=1.422 (95%CI: 0.205-9.870)
Probability of Survival
Time, Days
E
PFS
TP53/KRAS Co-mutated
TP53 MT
KRAS MT
10.5 vs. 10.5 vs. 15.3 months, p=0.245
Probability of Survival
Time, Days
F
OS
TP53/KRAS Co-mutated vs. TP53 MT
p=0.048*, HR=5.374
(95%CI: 1.015-28.44)
TP53/KRAS Co-mutated
TP53 MT
KRAS MT
24.1 vs. 39.7 vs. NR months, p=0.112
Probability of Survival
Time, Days
