# Supplementary material for: TP53 Mutation and Tumor‐Infiltrating Lymphocytes as Predictors of PD‐1/PD‐L1 Inhibitor Efficacy in PD‐L1‐High Advanced NSCLC
Source: Cancer Rep (Hoboken). 2026 Aug 13;9(8):e70648. doi: 10.1002/cnr2.70648 (PMC13473177; doi:10.1002/cnr2.70648)
Supplement: Supplementary file 2 [file CNR2-9-e70648-s002.pdf]

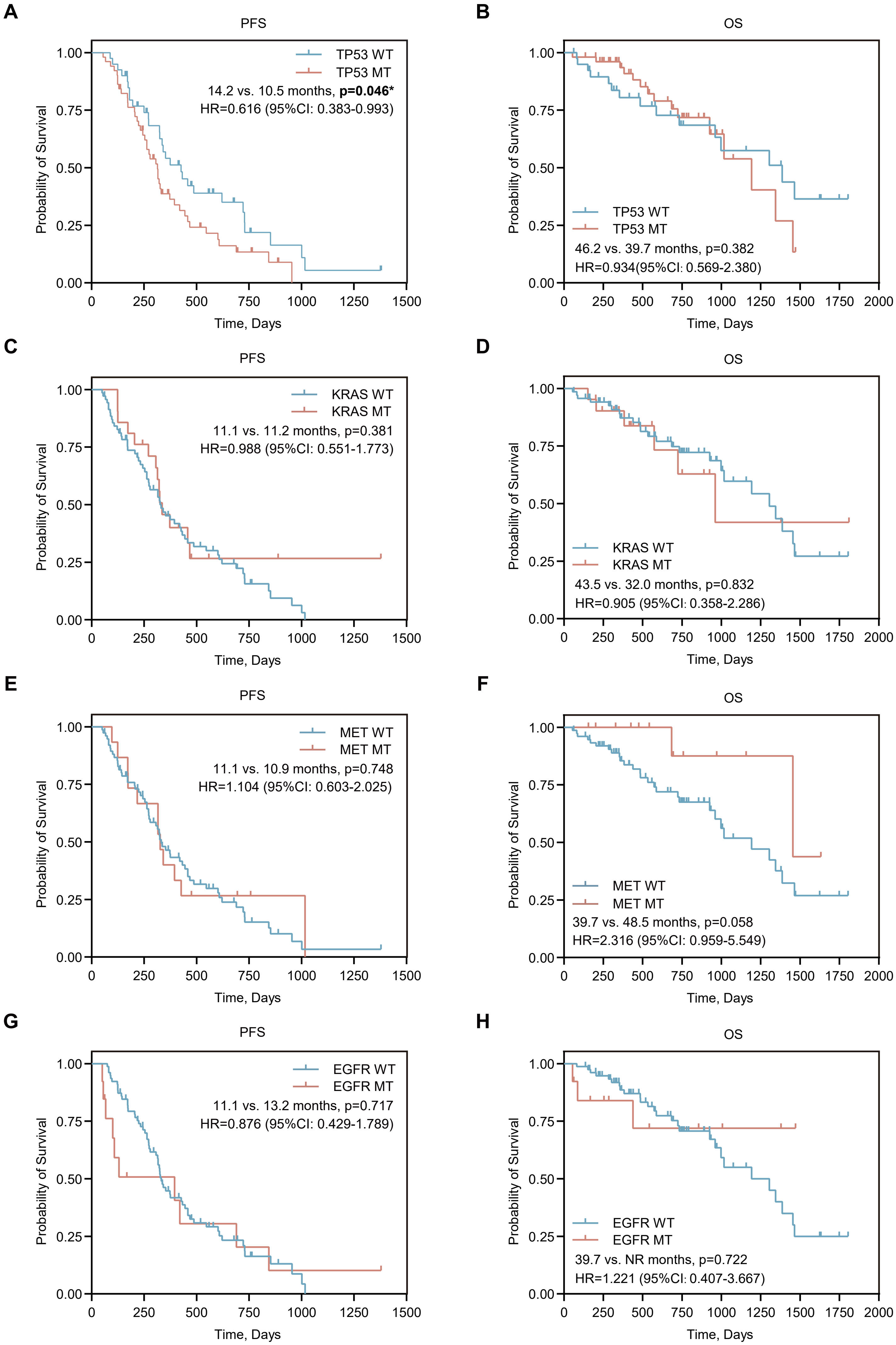
A
PFS
TP53 WT
TP53 MT
14.2 vs. 10.5 months, **p=0.046***
HR=0.616 (95%CI: 0.383-0.993)
Probability of Survival
Time, Days
B
OS
TP53 WT
TP53 MT
46.2 vs. 39.7 months, p=0.382
HR=0.934(95%CI: 0.569-2.380)
Probability of Survival
Time, Days
C
PFS
KRAS WT
KRAS MT
11.1 vs. 11.2 months, p=0.381
HR=0.988 (95%CI: 0.551-1.773)
Probability of Survival
Time, Days
D
OS
KRAS WT
KRAS MT
43.5 vs. 32.0 months, p=0.832
HR=0.905 (95%CI: 0.358-2.286)
Probability of Survival
Time, Days
E
PFS
MET WT
MET MT
11.1 vs. 10.9 months, p=0.748
HR=1.104 (95%CI: 0.603-2.025)
Probability of Survival
Time, Days
F
OS
MET WT
MET MT
39.7 vs. 48.5 months, p=0.058
HR=2.316 (95%CI: 0.959-5.549)
Probability of Survival
Time, Days
G
PFS
EGFR WT
EGFR MT
11.1 vs. 13.2 months, p=0.717
HR=0.876 (95%CI: 0.429-1.789)
Probability of Survival
Time, Days
H
OS
EGFR WT
EGFR MT
39.7 vs. NR months, p=0.722
HR=1.221 (95%CI: 0.407-3.667)
Probability of Survival
Time, Days
